# Neural Correlates of Social Inclusion in Borderline Personality Disorder

**DOI:** 10.3389/fpsyt.2018.00653

**Published:** 2018-12-03

**Authors:** Kathrin Malejko, Dominik Neff, Rebecca Brown, Paul L. Plener, Martina Bonenberger, Birgit Abler, Heiko Graf

**Affiliations:** ^1^Department of Psychiatry and Psychotherapy III, University Hospital Ulm, Ulm, Germany; ^2^Department of Child and Adolescent Psychiatry and Psychotherapy, University Hospital Ulm, Ulm, Germany

**Keywords:** borderline, depression, fMRI, social inclusion, social interaction

## Abstract

Humans engage in social interactions and have a fundamental need and motivation to establish and maintain social connections. Neuroimaging studies particularly focused on the neural substrates of social exclusion in healthy subjects (HC), borderline personality disorder (BPD), and major depression (MD). However, there is evidence regarding neural alterations also during social inclusion in BPD that we intended to elucidate in our study. Considering that patients with BPD often have comorbid MD, we investigated patients with BPD, and comorbid MD, patients with MD without BPD, and a sample of HC. By investigating these two clinical samples within one study design, we attempted to disentangle potential confounds arising by psychiatric disorder or medication and to relate neural alterations under social inclusion specifically to BPD. We investigated 48 females (15 BPD and MD, 16 MD, and 17 HC) aged between 18 and 40 years by fMRI (3T), using the established cyberball paradigm with social exclusion, inclusion, and passive watching conditions. Significant group-by-condition interaction effects (*p* < 0.05, FWE-corrected on cluster level) were observed within the dorsolateral (dlPFC) and dorsomedial prefrontal cortex (dmPFC), the temporo-parietal junction (TPJ), the posterior cingulate cortex (PCC), and the precuneus. Comparisons of estimated neural activations revealed that significant interaction effects were related to a relative increase in neural activations during social inclusion in BPD. In detail, we observed a significant increase in differential (social inclusion vs. passive watching) neural activation within the dmPFC and the PCC in BPD compared to both, MD and HC. However, significant interaction effects within the dlPFC and the TPJ could not specifically be linked to BPD considering that they did not differ significantly between the two clinical groups in *post-hoc* comparisons. Our study supports previous results on effects of social and inclusion in BPD, and provides further evidence regarding disorder specific neural alterations in BPD for brain regions associated with self-referential and mentalizing processes during social inclusion.

## Introduction

Humans are fundamentally motivated to achieve and maintain social relationships and social ties have a significant impact on well-being and mental health (1–3). Recent neuropsychological and social neuroscience mainly focussed on ostracism, social exclusion, and rejection, that occurs when an individual is excluded from a social interaction (3). Various neuroimaging studies investigated the underlying neural substrates of social exclusion and suggested two distinct but interconnected neural networks (4). Enhanced activations within the dorsal anterior cingulate cortex (dACC) and the anterior insula (aI) could be associated with social distress (2, 5–8), whereas increased neural activity within the dorsal (dmPFC) and ventral medial prefrontal cortex (vmPFC), the precuneus and the temporo-parietal junction (TPJ) were most likely related to self-referential mentalizing processes (9, 10).

Clinically, social rejection has been identified as a potential risk factor for several psychiatric disorders, particularly borderline personality disorder [BPD; (11, 12)], but also depression (13). Given the intense fear of loss or social rejection as a clinical core symptom of BPD, neural alterations within brain regions previously linked to social rejection seem plausible in this disorder. An early investigation using functional near infrared spectroscopy revealed increased neural activations in the medial prefrontal cortex (mPFC) during social exclusion in a small sample of BPD-patients (14).

Recent evidence also suggests an altered cerebral processing of social inclusion in BPD. Apart from the intense fear of loss, the self-perception of patients with BPD is characterized by negative belief of not being likable and the assumption that others are untrustworthy or will reject them (15–17). This core dysfunctional beliefs are self-maintaining, structure the patients' perception and interpretation of environmental stimuli and cause them to habitually react in ways that confirm their beliefs (15). In line with this, an early hypothesis called Festinger's theory of cognitive dissonance (18) assumed that an encountered mismatch between expectation and current experience is resolved by changing the perception to match the expectation. Domsalla et al. (19) investigated a sample of patients with BPD compared to healthy controls (HC) by functional magnetic resonance imaging (fMRI) using the established ball tossing paradigm “cyberball” (20) and focused also on social inclusion. Subjective feelings of rejection were significantly higher in BPD during the social inclusion condition. Moreover, increased neural activations were demonstrated during social inclusion within regions such as the dmPFC and the precuneus, that were previously observed during social exclusion and assigned to self-referential mentalizing in HC. These neural alterations in BPD during social inclusion might reflect the attempt to reduce such a cognitive dissonance and may further support the idea of a common altered neural processing of social interaction in this disorder (19).

Behavioral and neural alterations of social interaction have also been observed in major depression (MD). These findings may be of great relevance considering the high comorbidity of BPD and MD that may question the specificity of the findings in BPD (21). Apart from an increased rejection sensitivity in depressive patients (22, 23), neuroimaging studies associated emotional pain after social rejection with enhanced neural activations in the ACC, the amygdala, the aI and the ventrolateral prefrontal cortex (vlPFC) in MD compared to HC (5, 24, 25). Neural correlates of social inclusion were not investigated in MD by neuroimaging so far, but one electrophysiological study suggested a deficient processing of pleasurable social stimuli such as social inclusion compared to HC (26).

Thus, behavioral and neural alterations during social interaction situations were demonstrated for BPD and also MD, but previous studies focused more on social exclusion. Moreover, interpretations of these results comparing the two clinical groups separately to HC, may be confounded by further clinical characteristics such as comorbidity or medication. Although BPD-patients with current depressive episodes were excluded in a previous study (19), potential confounds may arise from still higher depression sumscores in this sample. In general, a comorbid depressive disorder *per se* may also bias the findings, considering the neural alterations during the processing of emotional and social stimuli even in remitted MD (27).

Motivated by these concerns, we now investigated a sample of BPD- and a clinical control group of MD-patients compared to HC within one study design. By including a clinical control group, we intended to control for effects of depression and medication. During fMRI, we used the established cyberball paradigm to warrant comparability with former studies in BPD and MD. Considering the previous hints on neural alterations under social inclusion in BPD as suggested by Domsalla et al. (19), we focused on this condition and hypothesized an increase in neural activation within previously identified brain regions related to self-referential and mentalizing processes such as the dmPFC, the TPJ and the precuneus. Moreover, we assumed an alteration in these regions under social inclusion specifically in BPD and intended to disentangle effects by comparisons with a clinical group diagnosed with MD without BPD with similar depressive symptoms and also under medication.

## Methods

### Subjects

We investigated a total of 48 females aged 18–40 years. Of those, 15 patients were diagnosed with BPD and 16 patients with major depressive disorder (MD). Seventeen healthy controls (HC) served as control group with no current or lifetime psychiatric diagnoses. Patient groups and HC were roughly matched for highest degree of education and age with however, higher mean age in the MD-group (see Table 1). At time of investigation, 13 of the 15 BPD-patients had a comorbid MD, two BPD-subjects were diagnosed with a remitted depression. Eight patients of the BPD-group met also criteria of posttraumatic stress disorder (PTSD) and 2 met criteria of dysthymia according DSM-IV. In the MD-group, two subjects met criteria of dysthymia according DSM-IV but had no other psychiatric diagnoses at the time of investigation and one had a history of remitted anorexia. Participants were recruited from inpatient and outpatient units of the Department of Psychiatry and Psychotherapy of the University Hospital Ulm. All 48 participants were right-handed according to the Edinburgh Handedness Inventory. Regular smoking cigarettes was reported from 3 of the MD-, 10 of the BPD- and 4 of the HC-group but was prohibited at least 2 h before fMRI-scanning. Participants with any severe medical disorder, epilepsy, substance use disorder and psychotic disorders were excluded from the study. Antidepressant medication was not interrupted, whereas the intake of sedative drugs was disrupted prior to scanning. All MD-patients took antidepressant medication of various kinds, one had a concomitant medication with topiramate, one with quetiapine and one with pregabalin, all of which were paused for at least 3 days before fMRI scanning, corresponding to 5 times the half-life of the substances. Twelve patients of the BPD-group took antidepressants and one was medicated with lithium. Further details on medication are provided in Table S1. All participants gave written informed consent prior to the study that was approved by the local ethical committee of Ulm University and conducted in accordance with the Declaration of Helsinki.

**Table 1:** Results of demographic and psychometric measurements in the healthy control (HC), borderline personality disorder (BPD) and major depression (MD) group.

	**HC**		**BPD**		**MD**		**One-way ANOVA**		Effect sizes (Hedge's g^*^) from ***post hoc*** **tests**		
	**Mean**	***SD***	**Mean**	***SD***	**Mean**	***SD***	**F (2.45)**	***p***	**HC vs. BPD**	**HC vs. MD**	**BPD vs. MD**
Age (years)	23.1	4.26	23.3	4.13	28.7	4.59	8.60	0.001	0.046	1.235^+^	1.202^+^
Education (school years)	10.8	1.70	10.4	1.64	10.8	1.68	0.23	0.798			
BDI	3.24	3.95	40.07	13.72	33.63	11.48	58.53	<0.001	3.661^+^	3.499^+^	−0.497
BIS total	57.76	7.88	70.00	8.52	65.25	8.15	9.17	<0.001	1.458^+^	0.912^+^	−0.555
BSL	0.21	0.25	2.45	1.28	1.46	0.92	24.77	<0.001	2.444^+^	1.835^+^	−0.870^+^
HFS	18.21	4.51	27.13	3.50	24.88	3.42	23.43	<0.001	2.136^+^	1.619^+^	−0.633
NTS belongings	14.65	4.06	9.33	4.05	13.94	5.09	6.60	0.003^*^	−1.279^+^	−0.151	0.972^+^
NTS self esteem	14.53	4.43	7.27	1.94	13.31	6.04	11.60	<0.001^*^	−2.024^+^	−0.226	1.293^+^
NTS meaningful existence	15.41	4.69	8.87	3.40	14.38	5.37	9.17	<0.001^*^	−1.541^+^	−0.200	1.185^+^
NTS control	12.18	3.52	6.27	1.16	8.56	2.66	19.90	<0.001^*^	−2.142^+^	−1.127^+^	1.074^+^
NTS total-score	2.84	0.70	1.59	0.40	2.51	0.88	13.73	<0.001^*^	−2.102^+^	−0.406	1.296^+^
RSQ	8.11	2.83	18.66	6.91	13.61	6.02	14.95	<0.001	1.996^+^	1.153^+^	−0.761^+^
**FEELINGS AFTER fMRI**											
Angry	1.18	0.64	2.29	1.14	1.63	0.72	6.71	0.003^*^	1.191^+^	0.646	−0.679^+^
Sad	1.35	0.70	2.14	1.03	1.88	1.02	2.99	0.060			
Happy	2.59	0.80	1.36	0.84	1.81	1.05	7.47	0.002^*^	−1.464^+^	−0.819^+^	0.459
Afraid	1.24	0.44	2.29	1.07	1.94	0.85	6.88	0.003^*^	1.282^+^	1.019^+^	−0.354
Frustrated	1.35	0.70	2.21	1.05	2.00	0.97	3.88	0.028^*^	0.952^+^	0.754	−0.203
Satisfied	2.59	0.62	1.43	0.94	1.81	0.91	8.07	0.001^*^	−1.439^+^	−0.983^+^	0.400
Helpless	1.53	1.07	2.50	1.16	2.00	1.26	2.67	0.081			
Inner tension	1.82	0.73	2.79	0.80	2.63	0.96	6.14	0.004^*^	1.239^+^	0.931^+^	−0.176

BDI, Beck Depression Inventory; BIS, Barratt Impulsiveness Scale; BSL, Borderline Symptom List; HFS, Hurt Feeling Scale; NTS, Need Threat Scale; RSQ, Rejection Sensitivity Questionnaire; numeric scales of “feelings after fMRI” range from 1 (very low) to 4 (very strong); SD, standard deviation; vs., versus; ANOVA, analysis of variance. To control for multiple comparison of the ANOVAs in dependent measures (NTS and feelings after fMRI); a false-discovery rate (FDR) correction was applied significant at p < 0.05 (FDR-corrected) to interfere significant main effects (

* ). For better demonstrational purposes, we provide measures of effect sizes (Hedges g*) for post hoc comparisons. Please note, positive values of effect sizes reflect higher scores for BPD when comparing HC vs. BPD, and higher scores for MD when comparing HC vs. MD and BPD vs. MD. Moreover, please consider the coding for the NTS-scale: scores range from 1 (very high distress) to 5 (no distress). (

+ *) denotes significant (p < 0.05) differences in post hoc comparisons (Newman Keuls). Readers are reminded that all state measures (NTS and feelings after fMRI) had been taken after the end of the whole cyber-ball paradigm*.

### Psychometric measurements

Clinical diagnoses of MD and BPD were verified by one of the study psychologists or physicians using the Structured Clinical Interview for DSM-IV [SCID-I and -II; (28)]. BPD symptom severity was assessed by the Borderline Symptom List [BSL-23; (29)] and total scores divided by the number of items served for further analyses. Current depressive symptoms were assessed by using the Beck Depression Inventory [second edition, BDI-II; (30)] in its German version (31). To assess impulsivity as personality trait we applied the Barratt Impulsiveness Scale in its 11th revision [BIS-11; (32), (33)], a self-reporting questionnaire that contains of 30 items, each rated on a 4-point Likert scale ranging from 1 (rarely/never) to 4 (almost always). Here, higher sumscores reflect higher trait impulsivity. Rejection sensitivity defined as a cognitive-affective processing disposition of anxious expectation, ready perception and overreaction to rejection cues was assessed by a German version of the Rejection Sensitivity Questionnaire [RSQ; (34), (35)]. Here, total scores were calculated according to (35) by multiplying scores from the subscale “anxiety or concern” with inverted scores of the “expectancy of acceptance” subscale divided by the number of items. Further, the Hurt-Feelings-Scale [HFS; (36)] was used to examine general sensitivity for social exclusion. This scale consists of eight questions concerning the sensitivity in social situations and each question is rated on a 5-point Likert-scale. Here, higher total scores correspond to higher sensitivity to social exclusion.

To assess distress after social exclusion elicited by the ball-tossing fMRI-paradigm, we used the established 20-item Need-Threat-Scale [NTS; (37)]. This scale comprises four subscales (“feeling excluded,” “low self-worth,” “meaningless existence,” “control”) with five items each. Each item was rated on a scale from 1 (not at all) to 5 (very much) and total scores were calculated by dividing the final result by 20, resulting in a range from 1 (very high distress) to 5 (no distress). A one-way analysis of variance (ANOVA) and *post-hoc* Newman Keuls tests were computed to analyze psychometric scales. To control for multiple comparisons of the ANOVAs in dependent measures such as the NTS and the feelings after fMRI (see below), a false-discovery rate (FDR) correction was applied at *p* < 0.05. In case F-values passed this FDR-corrected *p*-value, *post hoc* Newman Keuls tests were computed to explore between-group-differences motivating the main effect.

### fMRI paradigm

To examine social interactions under laboratory settings during fMRI and to warrant comparability with previous investigations, we used the well-established “cyberball” paradigm (20). Participants were instructed to take part in the virtual ball-tossing game with two other participants that were supposed to be in another room. In fact, participants played against the computer and all actions were pre-programmed (2, 20). Participants were represented by a hand at the bottom part of a screen, while the other players were represented by animated comic figures. The tasks consisted of three different conditions (“inclusion,” “exclusion,” and “passive watching” condition), each condition was applied once, each lasting around 2 min (60 throws). During “passive watching” condition, participants were asked just to watch the game and the computer controlled their character. In the second “inclusion” condition, participants had a random ball possession in one third of tosses. The “exclusion” session started with 10 throws of 30% randomized ball possession and then participants were excluded from the game for the remaining 50 throws. Information about the necessity of deception in this experiment and the real nature of the game was given in a debriefing session in verbal and written form after the assessment. After fMRI-scanning, subjective emotional experience regarding predefined indicated feelings (“angry,” “sad,” “happy,” “afraid,” “frustrated,” “satisfied,” “helpless,” and “inner tension”) was assessed on a visual rating scale ranging from 1 (very low) to 4 (very strong).

### fMRI data acquisition

Due to a scanner update during data acquisition, functional imaging data of the HC- and the BPD-group were obtained by a 3T MAGNETOM Allegra (Siemens, Erlangen, Germany) and those of the MD-group by a 3T MAGNETOM Prisma Scanner (Siemens, Erlangen, Germany). A T2^*^-sensitive gradient echo sequence was used for functional imaging with an echotime (TE) of 33 ms, a flip angle of 90°, a field of view (FOV) of 230 mm, and a slice thickness of 2.5 mm with an interslice gap of 0.5 mm. At a repetition time (TR) of 2,000 ms, 35 transversal slices were recorded with an image size of 64 × 64 pixels during the cyberball task. Anatomical high-resolution T1-weighted images (1 × 1 × 1 mm voxels) were acquired [band-width (BW) 130 Hz/Pixel, TR 2,500 ms, TI 1.1s, echotime (TE) 4.57 ms, flip angle 12°] for reasons of coregistration and normalization into standardized stereotactic space.

### fMRI-data analysis

Image pre-processing and statistical analyses were carried out using Statistical Parametric Mapping (SPM12, Wellcome Department, London, United Kingdom) with a random effects model for group analyses. Data from each session were pre-processed including slice-timing, realignment, and normalization into a standard template (Montreal Neurological Institute, MNI) with a spatial resolution of 2 × 2 × 2 mm^3^. Smoothing was applied with an 8-mm FWHM isotropic Gaussian kernel. Intrinsic autocorrelations were accounted for by AR (1) and low frequency drifts were removed via high-pass filtering (1/128s). For individual first level analyses, a general linear model was set up with the cyberball task modeled as three separate blocks of condition (passive watching, inclusion and exclusion) according to Eisenberger et al. (38, 39). Regressors representing the six motion parameters were integrated into the design matrix. After model estimation, beta-images representing the averaged, estimated magnitude of neural activation associated with passive watching, inclusion, and exclusion were computed for each participant and then submitted to random-effects group analysis. To verify whether our task had the desired effect, we calculated an F-test to examine the main effect of “condition” (passive watching, inclusion, exclusion) in HC (see Supplementary Material). For second level group analyses, we computed a 3 × 3 ANOVA model (omnibus *F*-test) with the two factors “group” (HC, BPD, MD) and “condition” (passive watching, inclusion, exclusion) that included first level contrasts for the three ball tossing conditions for each of the three groups. Between-group-differences for the different condition contrasts were inferred with an appropriate group-by-condition interaction F-test with a significance level of *p* < 0.001 at the voxel-level and at least 90 contiguously significant voxels corresponding to family-wised error (FWE) correction of *p* < 0.05 on cluster level. This specific number of 90 voxels was computed with the SPM extension “Corr-ClussTh.m” (script provided by Thomas Nichols, University of Warwick, United Kingdom, and Marco Wilke, United Kingdom, University of Tübingen, Germany; https://warwick.ac.uk/fac/sci/statistics/staff/academic-research/nichols/scripts/spm/spm8/corrclusth.m). In case of a significant main effect, single tailed t-interaction contrasts were computed to explore which of the group difference motivated the significant interaction *F*-test. Based on previous studies and our hypothesis, we focused on differential neural activation under social inclusion vs. passive watching as motivated by Domsalla et al. (19). Since data collection of the BPD- and HC-group occurred at a different scanner than for the MD-group, scanner type was included as a covariate in all further analyses. Considering that patients within the MD-group were significant older than BPD-patients and HC, we additionally included age as a second covariate. In case of significant neural alterations in BPD during social inclusion compared to both, HC and MD, we also examined whether these neural activations in BPD were associated with different individual extents of psychopathological features in the BPD group. Thus, we computed correlation analyses between significant differential cluster fMRI parameter estimates and scores from those psychometric scales that also differed significantly between BPD and both control groups, MD and HC.

## Results

### Psychometric measurements

According to the clinical diagnoses of the participants, significantly higher BDI- and BIS-sumscores were observed in both, the BPD- and MD-group compared to HC. Despite a trend of higher sumscores in BPD, the two clinical goups did not differ significantly regarding depressive symptom severity and impulsivity. Higher overall BSL-scores were observed in the BPD- than in the MD-group that, however, revealed still higher scores than HC. General sensitivity for social exclusion examined on the one hand by the HFS revealed significantly higher overall scores in BPD and MD compared to HC whereas the BPD- and MD-group did not differ from each other, although, BPD patients again showed slightly higher values. As another measure, the RSQ was used to examine trait rejection sensitivity that was statistically most prominent in BPD compared to HC and MD but also higher in MD compared to HC.

Regarding the NTS, the ANOVA revealed a significant effect for the factor group on total and subscale scores. In detail, NTS total scores and subscores concerning the feeling of “belonging” “self-esteem,” “meaningful existance,” and “control” were lower in BPD compared to MD and HC. However, the MD-group significantly differed solely from HC in terms of the experience of less control. Regarding the subjective ratings of individual feelings after the entire fMRI-cyberball paradigm, patients within BPD- and the MD-group felt significantly less “happy” and “satisfied” compared to HC. Moreover, patients with BPD felt more angry, afraid, frustrated and inner tension compared to HC, whereas they differ from MD-patients solely by feeling more anger. Total scores and analyses of psychometric measurements are summarized in Table 1.

### fMRI results

#### Main effect of condition

Before testing the main hypothesis as outlined above, we made use of the data from HC to test whether the present paradigm yielded condition effects in correspondence with those reported previously. In this analysis, we found significant (*p* < 0.05 FWE-corrected at cluster level) neural activations within the pregenual anterior cingulate cortex (pgACC), the parahippocampus, the precuneus, the precentral gyrus, the insula, and a cluster comprising the thalamus, the putamen and the right amygdala, i.e., in regions that were previously described in studies using the same paradigm (40–44). More details on this analysis and corresponding results are provided in Supplementary Material (Table S2 and Figure S1).

#### Interaction effect of group and ball tossing condition

A 3 × 3 ANOVA revealed significant (*p* < 0.001, *k* > 90 voxels corresponding to FWE-correction on cluster level) group-by-condition interaction effects within the dorsolateral (dlPFC, BA46) and dorsomedial prefrontal cortex (dmPFC, BA9), the temporo-parietal junction (TPJ, BA22), the posterior cingulate cortex (PCC, BA23) and the precuneus (BA7). Interestingly, against expectation we did not observe significant interaction effects within the anterior cingulate cortex (ACC) and anterior insula (aI) at the given threshold of statistical inference, despite the fact that these regions have been repeatedly reported in previous studies conducted with the same paradigm (44). We therefore explored this phenomen abandoning the cluster size thresholds of 90 voxels and observed trendwise group differences in the pregenual anterior cingulate cortex [pgACC; MNI (x/y/z): 6/46/-4; Z: 3.70; cluster size: 41 Vx] and the anterior insula [aI; MNI (x/y/z): −24/28/12; Z: 4.34; cluster size: 86 Vx].

**Figure 1:**
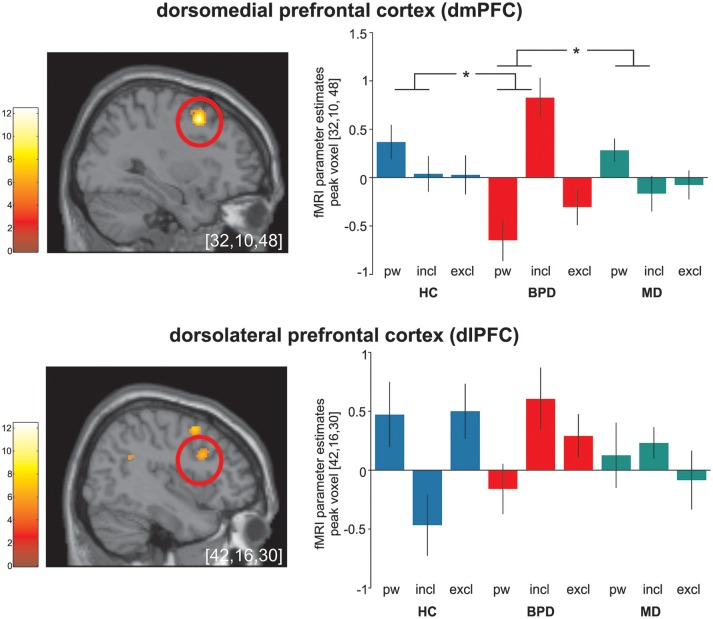
Significant (*p* < 0.05 FWE-corrected on cluster level) group-by-condition interaction effects within the dorsomedial prefrontal cortex (dmPFC) and the dorsolateral prefrontal cortex (dlPFC) during the cyberball paradigm in borderline personality disorder (BPD, red), major depression (MD, green) and healthy controls (HC, blue). *Post hoc* computed single tailed t-interaction contrasts revealed that differential (social inclusion vs. passive watching) neural activations within the dmPFC were related to BPD and differed significantly from those under MD and HC. Regarding neural activations within the dlPFC, on might assume a similar activation pattern in BPD and MD in contrast to those in MD. However, it is of note that *post-hoc* comparisons did not differ between groups. The brain slide depicts significant neural activations revealed from the 3 × 3 ANOVA. Bar charts show fMRI parameter estimates from peak voxel activation within the dmPFC and the dlPFC with standard error of the mean during passive watching (pw), social inclusion (incl.) and exclusion (excl.) conditions. ^*^Depicts significant between group differences in differential (social inclusion minus passive watching) neural activation (see also Table 2); MNI-coordinates [x,y,z] in mm.

*Post hoc* comparisons on differential neural activations were computed to explore differences between groups and revealed significant enhanced neural activities within the dmPFC (BA9), the TPJ (BA22), and the PCC (BA23) in BPD compared to HC under social inclusion vs. passive watching conditions. However, neural activations within the left precunes (BA7) did not survive corrections for multiple comparisions at the cluster level. Considering that these neural activations could also relate to MD, we explored neural activations between the two clinical groups and revealed that differential (social inclusion vs. passive watching) neural activations within the dmPFC (BA9), the PCC (BA23), and the precuneus (BA7) were enhanced in BPD even when compared to MD (*p* < 0.05; FWE-corrected at cluster-level). Notably, differential neural activations (social inclusion vs. exclusion) did not reveal significant group differences. Detailed results on significant interaction effects are depicted in Table 2 and Figure 1.

**Table 2:** Significant (*p* < 0.001, *k* > 90 Vx corresponding to FWE-correction on cluster level) whole brain group-by-condition interaction effects and *post-hoc* analyses (*p* < 0.001, *k* > 150 Vx corresponding to FWE-correction on cluster level) comparing differential (inclusion vs. passive watching condition) neural activations between groups (HC, healthy controls; BPD, borderline personality disorder; MD, major depression) during the fMRI cyberball paradigm.

**BA**	**Anatomic label**	**Side**	**MNI**			**Z**	**Cluster size**	***Post hoc*** **analyses** ***(p)***	
		**L/R**	**x**	**y**	**z**		**NV**	**BPD>HC**	**BPD>MD**
								**incl.>pw**	**incl.>pw**
46	Dorsolateral prefrontal cortex (dlPFC)	L	−44	28	34	4.34	338		
		R	42	16	30	3.75	117		
9	Dorsomedial prefrontal cortex (dmPFC)	R	32	10	48	5.37	451	0.002	0.001
22	Temporoparietal junction (TPJ)	R	50	−52	24	4.31	224	0.013	
23	Posterior cingulate cortex (PCC)	L	−2	−20	30	3.63	225		
		R	6	−26	32	4.18	#	0.023	0.041
7	Precuneus	L	−8	−70	42	4.50	355		0.003
		R	12	−58	32	4.17	129		

*BA, Brodman area; L, left; R, right; NV, number of voxels; MNI, Montreal Neurological Insitute (x-, y-, z-coordinates are provided in mm), Z, Z-value; incl., inclusion condition; pw, passive watching; #,activation is part of the cluster above*.

#### Correlation analyses

As mentioned above and outlined in Table 2, there were two brain regions only fulfilling the criterion to bear significant between-group differences when comparing differential (social inclusion vs. passive watching) neural activations in BPD against both control groups: right dmPFC and PCC. To examine whether these neural alterations within BPD patients were associated with psychopathological features, we extracted parameter estimates of differential neural activation from these clusters and computed correlation analyses with individual BSL- and RSQ-scores considering that these were the only two psychometric scales that also fulfilled the criterion to show significant between group differences of BPD compared to both, HC and MD (see Table 1).

Within BPD patients, we found a significant correlation between BSL-scores and differential neural activations within the right dmPFC (*r* = 0.56; *p* = 0.015) and the right PCC (*r* = 0.52; *p* = 0.023), indicating that higher borderline symptom severity was associated with higher neural activations during social inclusion vs. passive watching. Regarding RSQ-scores, we found no significant correlations with differential neural activations in these two regions.

Of note, we refrained from correlation analyses between neural activations and emotional reactions to the cyberball task (NTS and feelings after fMRI) considering that these scales were presented after the whole fMRI-paradigm instead of after each condition (see also Limitations section).

## Discussion

We investigated two clinical groups of patients, one with BPD and comorbid MD and the second with MD without BPD and compared against healthy controls (HC) using the established cyberball paradigm and fMRI to further elucidate previous reports on neural alterations under social inclusion in BPD. By the inclusion of these two clinical samples in one study design, we intended to control for potential confounds arising from depression, and to disentangle more disorder-specific neural patterns in brain regions relevant for social interaction in BPD. Psychometric measures beyond fMRI revealed similarly high general rejection sensitivity in both, BPD and MD compared to HC, though rejection sensitivity as a personality trait, and state markers of social distress and negative feelings after the cyberball task were most prominent in BPD. From fMRI data, we observed significant group-by-condition interaction effects within the dlPFC, the dmPFC, the TPJ, the PCC and the precuneus and thus in brain regions that were previously observed in studies conducted with the same paradigm. Notably, significant interaction effects in most of these regions were mainly determined by enhanced neural activations during social inclusion vs. passive watching in BPD. Considering that BPD-patients in our study also had relevant comorbid depressive symptoms, particularly enhanced neural activations within the dmPFC and the PCC were specifically related to BPD but not MD, as revealed by *post-hoc* comparisons between BPD- and MD-patients under social inclusion vs. passive watching. Here, correlation analyses revealed that higher neural activations within the right dmPFC and PCC under social inclusion in BPD were associated with higher individual symptom severity as measured by the BSL. However, neural activations under social inclusion nor exclusion did not differ between MD and HC.

The higher general sensitivity for social exclusion revealed by the HFS in BPD and MD compared to HC is in line with previous investigations in BPD (34, 35) and MD (22, 23). Beyond this comparable sensitivity regarding social exclusion, rejection sensitivity as a personality trait as measured by the RSQ was pronounced in BPD. Moreover, negative emotions after fMRI such as feeling angry, afraid, frustrated, and inner tension were significant higher in patients with BPD than in HC, whereas they differ from MD-patients solely by feeling more anger. In line with this, positive feelings were significantly less pronounced in BPD compared to both, MD and HC. This is in accordance with various studies using the same fMRI-paradigm that reported higher subjective feelings of social exclusion in BPD (19, 35, 45) that are even evident irrespective of the ball tossing condition and thus also during social inclusion (14, 19, 35, 45).

On a neural activation level, significant group-by-condition interaction effects were observed within the dlPFC, the dmPFC, the TPJ, the PCC and the precuneus. An involvement of these brain regions is in accordance with other investigations conducted with the same ball tossing paradigm (9, 10, 41). However, in these previous studies, brain regions were particularly reactive to social exclusion in HC while here, we investigated a specific reactivity to social inclusion in BPD. Accordingly, *post-hoc* comparisons on significant differential neural activations contrasting social inclusion vs. passive watching in depressed BPD compared to both, HC and MD, enabled us to determine potential disorder-specific neural alterations in BPD. In line with our initial hypthesis, a specific increase in neural activations during social inclusion was identified within the dmPFC and the PCC, and thus in regions that have been related to emotional conflict monitoring and self-referential mentalizing. Considering the functional division of the medial prefrontal cortex (46), the dorsal parts as found in our study are thought to encode the appraisal of emotional conflicts and action monitoring in social cognition tasks (19, 47, 48). In addition, the dmPFC has been critically involved in self-referential mentalizing about social knowledge (9, 10, 49, 50). Thus, enhanced self-referential processing under social inclusion in BPD may explain this activation pattern. This idea is further supported by a significant increase in neural activation within the PCC and, after applying an uncorrected statistical threshold, also within the precuneus. These regions are supposed to form a core mentalizing network implicated in social situations (51, 52). However, enhanced neural activations in these regions have been generally observed during the experience of social exclusion in healthy subjects (2, 53). Indeed, considering the typical internal belief of BPD patients that others will reject them (17), social cues such as social inclusion might conflict with internal assumptions. Increased neural activations in these regions may reflect these conflicts that in turn facilitate mentalizing processes to perceive or interpret intentions of the other (54–57). Our results are further in accordance with a recent investigation in BPD (19) that demonstrated increased neural activations within the dmPFC and the precuneus in BPD mainly under social inclusion compared to passive watching. Notably and in line with that study, neural increases in these regions were particularly pronounced when contrasting social inclusion to passive watching as compared to exclusion. Moreover, our results are in accordance with electrophysiological studies that demonstrated an increased P3 amplitude in BPD compared to HCs in social inclusion conditions (58, 59).

Significant interaction effects were further observed within the dlPFC and the TPJ. Also here, neural activations were mainly increased under social inclusion in BPD and MD, but it is of note that these alterations did not differ significantly between the two clinical groups or from HC. However, one might assume a similar activation pattern within the dlPFC in BPD and MD in contrast to HC. Neural activations within the dlPFC were previously associated to social cognition (60). Together with the ACC, the dlPFC has been linked to an internal “alarm system” to interpret incongruent events that violate social expectancies (61) whereas neural activations within the TPJ were consistently associated to the reorientation to salient stimuli and self-referential mentalizing in social cognition (61–63). Moreover, there are empirical findings that the central executive network as represented by the dlPFC and the TPJ response to relevant social and salient stimuli (64). However, taking into account that neural activations in these regions did not differ between groups, they may represent a mere general neural alteration during social inclusion in both clinical samples rather than a disorder-specific neural alteration in BPD.

Considering that social interaction has a profound effect on emotion, we expected neural alterations also within the anterior cingulate cortex (ACC) and the anterior insula that have been associated to a recruitment of affective components during the cyberball paradigm (41). However, interaction effects in these regions were not evident under the conservative statistical threshold. Neural activations within these regions were mainly related to social distress in HC (2, 5–8). Despite not significant after correction for multiple comparisons in our study, neural activations within the anterior insula and the pgACC were mainly enhanced under social inclusion in BPD. Considering a dorsal-cognitive and ventral-emotional functional dissociation within the ACC, neural activations in ventral parts of the ACC as within the pgACC, has been linked to emotion evaluation (41, 43) and saliency (65–67) and fits with our interpretation of enhanced reactivity of brain areas associated with emotional conflict monitoring and self-referential mentalizing during social inclusion in BPD. However, due to the lack of significant interaction effects, this interpretation remains speculative and is a subject of further research.

## Limitations

While our results provide a potential further evidence regarding disorder-specific neural alterations in BPD under social inclusion, several limitations have to be considered. The intensity regarding emotional reactions corresponding to our task were assessed by psychometric measures (NTS and “feelings after fMRI”) after the whole fMRI-paradigm but not separately after passive watching, inclusion, and exclusion. This limits the interpretability of our behavioral results regarding emotional reactions to each of the three task conditions and in particular to social inclusion. A further limitation may arise by the design of the task with an unbalanced ordering of conditions and the fact that subjects may not realize when exactly they are rejected during the exclusion condition. However, this issue should not have confounded our results regarding neural alterations under social inclusion, and the task was used in its original design to warrant comparability with previous investigations. Due to a scanner update during data acquisition, MD-subjects were investigated with a different fMRI-scanner as BPD and HC. In line with the evidence for robust and reliable patterns of fMRI-activations even when assessed by scanner of different manufactures (68, 69), we found no indication for systematic differences in image quality between both MR scanners, in particular, as the same manufacturer, same field strength and same acquisition parameters were applied. Nevertheless, we cannot entirely exclude potential confounds by different scanners, in particular regarding group comparisons relative to MD.

Consistent with the high prevalence of comorbid PTSD in BPD (70–74), 8 subjects in our BPD-sample met also criteria of PTSD. Thus, we cannot rule out potential effects of PTSD-symptoms on our results. This would have required the inclusion of a third clinical sample with PTSD but without BPD. However, our findings regarding neural alteration during social inclusion in BPD are in line with other fMRI-investigations in BPD without PTSD (19, 58, 59). To support comparability of medication, we investigated the two clinical samples under stable antidepressant medication that primarily facilitated monoaminergic, catecholaminergic neurotransmission. However, medication may have potentially altered neural activations relevant for emotion processing and saliency [e.g., (75)]. Considering that our observations regarding neural alterations during social inclusion in BPD were also found in studies conducted in BPD without medication (19), confounds arising by these antidepressants may be limited to comparisons between the two clinical groups. Thus, our results particularly relative to MD await empirical replication.

## Conclusion

We investigated patients with BPD and comorbid MD and patients with MD without BPD and compared them to HC during fMRI and an established version of the cyberball paradigm. Based on previous findings, we focused on neural alterations under social inclusion in BPD. By investigating these two clinical groups within one study design, we intended to control for potential confounds arising by depression and medication and to disentangle more disorder-specific neural alterations of social interaction in BPD. We observed significant group-by-condition interaction effects within the dlPFC, the dmPFC, the TPJ, the PCC and the precuneus. Notably, by contrasting neural activations in BPD compared to both, MD and HC, we demonstrated potential disorder-specific neural alterations within the dmPFC and the PCC in BPD under social inclusion and could associate them with individual borderline symptom severity. Considering the contribution of these brain regions to emotional conflict monitoring, self-referential processes and mentalizing, these alterations seem plausible considering the frequent internal assumption in BPD that others will reject them. Although significant interaction effects were also observed within the dlPFC and TPJ, these activations did not significantly differentiate BPD- from MD-patients in our study, thus, reflecting a mere general neural alteration in clinical samples with high rejection sensitivity. Our results provide further evidence regarding a disorder-specific neural alteration of social interaction that is predominantly determined by social inclusion.

## Author contributions

HG and KM contributed substantially to the work, analyzed and interpreted the data, and drafted the manuscript. BA, RB, and PP contributed substantially to the work, designed the study, interpreted the data, and revised the manuscript critically for important intellectual content. MB, DN, and RB obtained the data. All the authors approved the final version to be published and agreed to be accountable for all aspects of the work in ensuring that questions related to the accuracy or integrity of any part of the work are appropriately investigated and resolved.

### Conflict of interest statement

The authors declare that the research was conducted in the absence of any commercial or financial relationships that could be construed as a potential conflict of interest.
