# Supplementary material for: Neural Correlates of Social Inclusion in Borderline Personality Disorder
Source: Front Psychiatry. 2018 Dec 3;9:653. doi: 10.3389/fpsyt.2018.00653 (PMC6287007; doi:10.3389/fpsyt.2018.00653)
Supplement: Supplementary file 1 [file Data_Sheet_1.docx]

**Supplementary material**

**Neural correlates of social inclusion in borderline personality disorder**

Kathrin Malejko^1*^, Dominik Neff^1^, Rebecca Brown^2^, Paul L. Plener^2^, Martina Bonenberger^2^, Birgit Abler^1^, Heiko Graf^1^

^1^ Dept. of Psychiatry and Psychotherapy III, University Hospital Ulm, Germany

^2^ Dept. of Child and Adolescent Psychiatry and Psychotherapy, University Hospital Ulm, Germany

**Methods**

**Table S1:** Antidepressive medication in patients with borderline personality disorder (BPD) and major depression (MD). None of the healthy controls (HC) took any medication.

n=number of patients.

| *antidepressive medication* | *BPD (n)* | *MD (n)* |
| --- | --- | --- |
| sertraline |  | 6 |
| fluoxetine | 3 | 1 |
| escitalopram |  | 2 |
| bupropion |  | 1 |
| venlafaxine |  | 2 |
| trimipramine | 1 |  |
| doxepin | 1 |  |
| tranylcypromine | 1 |  |
| escitalopram + bupropion |  | 1 |
| sertraline + mirtazapine | 4 | 2 |
| fluoxetine + mirtazapine |  | 1 |
| venlafaxine + bupropion | 2 |  |

**Results**

*Main effect of condition*

To verify whether our task had the desired effect, we computed an F-test to examine the main effect of ‘condition’ (passive watching, inclusion, exclusion) in HC at a significance level of p<0.001 at the voxel-level and at least 115 contiguously significant voxels, corresponding to family-wised error (FWE) correction of p<0.05 on the cluster level. This specific number of 115 voxels was determined by using the SPM extension “Corr-ClussTh.m” (script provided by Thomas Nichols, University of Warwick, United Kingdom, and Marco Wilke, United Kingdom, University of Tübingen, Germany; https://warwick.ac.uk/fac/sci/ statistics/staff/academic-research/nichols/scripts/spm/spm8/corrclusth.m).

In this analysis we could confirm significant neural activations that were previously described in studies using the same paradigm ([Sebastian et al., 2011](#_ENREF_3);[Eisenberger, 2012](#_ENREF_1);[Nishiyama et al., 2015](#_ENREF_2);[Wagels et al., 2016](#_ENREF_4);[Wang et al., 2017](#_ENREF_5)). In detail, we revealed significant neural activations of the pregenual anterior cingulate cortex (pgACC), the parahippocamupus, the precuneus, the precentral gyrus, the insula, and a cluster comprising the thalamus, the putamen and the right amygdala (see **table S2** and **figure S1**).

**Table S2:** Significant (p<0.001, k>115 Vx corresponding to p<0.05 FWE-correction on cluster level) main effect of ‘condition’ in healthy controls during the cyberball paradigm.

BA=Brodman area; L=left; R=right; NV=number of voxels; MNI=Montreal Neurological Insitute (x-, y-, z-coordinates are provided in mm), Z=Z-value; # indicates different anatomical regions within the same statstically significant clsuter

| *BA* | *Anatomic label* | *side* | *MNI* | | | *Z* | *cluster size* |
| --- | --- | --- | --- | --- | --- | --- | --- |
|  |  | *L/R* | *x* | *y* | *z* |  | *NV* |
|  | pgACC | R | 6 | 44 | -4 | 3.81 | 171 |
|  |  | L | -8 | 48 | 2 | 3.60 | # |
| 37 | Gyrus parahippocampalis | R | 30 | -40 | -10 | 6.25 | 519 |
|  |  | L | -30 | -46 | -10 | 5.57 | 1114 |
|  | Precuneus | L | -12 | -62 | 20 | 4.91 | # |
| 3 | Gyrus precentralis | R | 36 | -18 | 40 | 4.65 | 232 |
| 13 | Insula | R | 34 | -10 | 22 | 4.32 | 381 |
|  | Thalamus | R | 4 | 4 | 10 | 4.64 | 1065 |
|  | Putamen | R | 22 | 6 | -10 | 4.78 | # |
|  |  | L | -12 | 12 | -4 | 4.43 | # |
|  | Amygdala | R | 20 | -4 | -16 | 2.97 | # |

**Figure S1:** fMRI-parameter estimates extracted from three exemplary regions (A: pregenual anterior cingulate cortex, pgACC; B: right amygdala; C: right thalamus) that revealed a significant main effect of ‘condition’ during the cyberball paradigm in healthy controls.

pw=passive watching; incl=inclusion condition; excl=exclusion condition; MNI-coordinates of peak voxels are provided in squared brackets in mm;


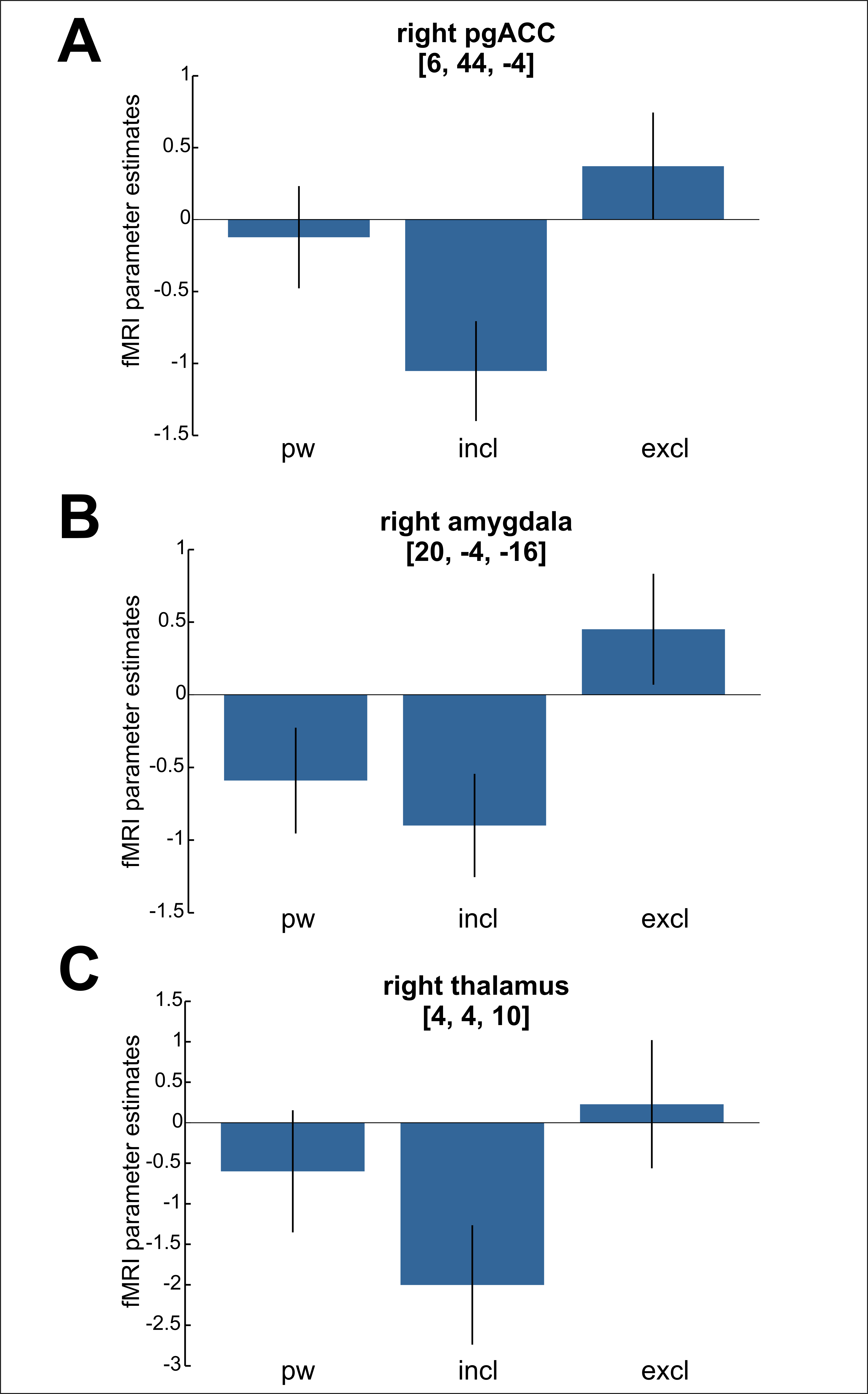


**References**

Eisenberger, N.I. (2012). The pain of social disconnection: examining the shared neural underpinnings of physical and social pain. *Nature reviews. Neuroscience* 13**,** 421-434.

Nishiyama, Y., Okamoto, Y., Kunisato, Y., Okada, G., Yoshimura, S., Kanai, Y., Yamamura, T., Yoshino, A., Jinnin, R., Takagaki, K., Onoda, K., and Yamawaki, S. (2015). fMRI Study of Social Anxiety during Social Ostracism with and without Emotional Support. *PloS one* 10**,** e0127426.

Sebastian, C.L., Tan, G.C., Roiser, J.P., Viding, E., Dumontheil, I., and Blakemore, S.J. (2011). Developmental influences on the neural bases of responses to social rejection: implications of social neuroscience for education. *NeuroImage* 57**,** 686-694.

Wagels, L., Bergs, R., Clemens, B., Bauchmuller, M., Gur, R.C., Schneider, F., Habel, U., and Kohn, N. (2016). Contextual exclusion processing: an fMRI study of rejection in a performance-related context. *Brain imaging and behavior*.

Wang, H., Braun, C., and Enck, P. (2017). How the brain reacts to social stress (exclusion)-a scoping review. *Neuroscience and biobehavioral reviews*.
